# Synthesis and applications of MANs/poly(MMA-co-BA) nanocomposite latex by miniemulsion polymerization

**DOI:** 10.1098/rsos.170844

**Published:** 2017-11-01

**Authors:** Sheng Gong, Huayao Chen, Xinhua Zhou, Sundaram Gunasekaran

**Affiliations:** 1 School of Chemistry and Chemical Engineering, Zhongkai University of Agriculture and Engineering, Guangzhou, Guangdong 510225, People's Republic of China; 2 Department of Biological Systems Engineering, University of Wisconsin–Madison, Madison, WI 53706, USA

**Keywords:** antimony-doped tin oxide, nanocomposite, core-shell structure, miniemulsion polymerization, spectrally selective films

## Abstract

We have synthesized core-shell structured 3-methacryloxypropyltrimethoxysilane (MPS) functionalized antimony-doped tin oxide nanoparticles (MANs)–poly(methyl methacrylate-co-butyl acrylate) (PMMA-co-BA, PMB) nanocomposite latex particles via miniemulsion polymerization method. Polymerizable anionic surfactant DNS-86 (allyloxy polyoxyethylene(10) nonyl ammonium sulfate) was first introduced to synthesize core-shell nanocomposite. The morphologies of synthesized MANs and MANs/PMB latex nanocomposite particles were studied with transmission electron microscopy, which revealed particles, on average 70 nm in size, with a core-shell structure. Owing to the uniformity and hydrophobicity of MANs, the MANs-embedded PMB latex nanocomposite can be tailored more precisely than other nanoparticles-embedded nanocomposites. Films incorporating 10 wt% of MANs in the MAN/PMB latex nanocomposite exhibit good transmittance in the visible region, and excellent opacity in the near infrared region. The MANs/PMB nanocomposite film also appears suitable for heat insulation applications.

## 1. Introduction

Nowadays, considerable attention is given to transparent conductive oxides (TCOs) due to their potential applications in fields such as optoelectronics, displays, solar cells etc. [1–4]. TCOs are excellent electrical conductors; they are highly transparent in the visible range and provide heat shield in the near infrared (NIR) region of solar radiation. Antimony-doped tin oxide (ATO) nanoparticles (NPs) are fairly transparent and relatively inexpensive, and hence are used as transparent electrodes in electrochemistry [1,2] or as substrates for electrode deposition [3] in solid-state devices for photovoltaic and optoelectronic applications [4–6], and/or as spectrally selective coatings [5,7]. There is growing interest in extending the ATO thin films to improve their optical and electrical properties. To achieve this, the effects of the method of preparation and uniformity of coating of dopants such as antimony (Sb) are being investigated. Several methods developed so far include sol–gel [8], chemical vapour deposition (CVD) [9], magnetron sputtering [10], pulsed laser deposition (PLD) [11] and spray pyrolysis [12]. However, the need for vacuum equipment or high-temperature processes usually required in these methods limits the industrial applications of ATO, although ATO is more available than indium tin oxide (ITO) and thus its use is generally more cost-effective.

Low emissivity glass (low-E glass) has a high transmittance in the visible region and high reflectance in the far infrared region [13]. Low-E glass is widely used in energy-efficient windows, because it can block not only the NIR radiation (known as source of heat) from penetrating the glass, but also the emission of far infrared radiation from inside the building. Deposition techniques used for low-E glass include CVD [9], sputtering [10], PLD [11], spray pyrolysis [12], etc. These processes usually need high temperatures or high electron energies or large vacuum equipment. Therefore, their disadvantages are also obvious: low productivity, high cost and difficult to apply on existing architectural glass [14].

A new class of polymeric materials, which combines the properties of inorganic particles with the processability and flexibility of organic polymer matrices, will have excellent properties, such as high mechanical strength and thermal stability. The possibility of developing polymer nanocomposites affords achieving ATO NPs with good optical, thermal and barrier properties. There are many ways to fabricate such polymer/inorganic nanocomposites, including via mechanical mixing [15], melt mixing [16] and *in situ* controlled radical polymerization etc. [17]. Among these, the method of synthesizing a nanocomposite made of inorganic particles surrounded by polymers, to achieve good dispersion of the inorganic compound and to increase interfacial adhesion between the polymer and the mineral, is very appealing.

Miniemulsion polymerization is a promising method for preparing nanoscale latex particles [18–22]. Materials fabricated by miniemulsion polymerization are used as adhesives [23]; anti-reflective- [24], anticorrosive- and UV-resistant-coatings [25,26]; textile pigments [27]; polymer fillers [28,29]; ultrabright fluorescent NPs [30] and so on. The morphology of these nanocomposite particles are core-shell [31] and raspberry-like [32,33], depending on the size of the inorganic particles and their surface chemistry.

Herein, we report the preparation of a transparent thermal insulation aqueous latex with irregular distribution of ATO NPs by miniemulsion polymerization. As a result, the MPS-functionalized ATO NPs (MANs)-embedded PMB latex nanocomposite can be tailored more precisely than other NPs-embedded nanocomposites [34]. We first introduce a polymerizable anionic surfactant DNS-86 (allyloxy polyoxyethylene(10) nonyl ammonium sulfate) to synthesize highly stable, transparent core-shell nanocomposite with infrared shielding properties via miniemulsion polymerization in the presence of ATO, a key filler, which was functionalized with reactive 3-methacryloxypropyl trimethoxysilane (MPS) to promote intercalation of water-insoluble monomers methyl methacrylate (MMA) and butyl acrylate (BA). The distribution and degree of aggregation of MANs in the nanocomposite film are controlled by varying the ATO content and the inorganic–organic hybrid polymerization process.

## 2. Experimental

### 2.1. Materials

ATO NPs, of mean particle size approximately 20–25 nm (Sb/Sn = 1/9 (n/n)), were purchased from Huzheng Nanotechnology Co. MPS was supplied by Dow Corning (China) Holding Co. Anhydrous ethanol and ammonium hydroxide (28%) were from Guangzhou Donghong Chemical Reagent Co. Sodium lauryl sulfate (SLS) and sodium bicarbonate (NaHCO_3_) were from Guangzhou Chemical Reagent Co. Co-stabilizer hexadecane was from Aldrich. MMA and BA were purchased from Guangzhou Kermel Chemical Reagent Co. Azobisisobutyronitrile (AIBN) (Guangzhou Chemical Reagent Co.) was recrystallized in anhydrous ethanol. DNS-86, a polymerizable surfactant, was purchased from Guangzhou ShuangJian Chemical Co. Glass slides (25.4 mm × 76.2 mm × 1.2 mm) and glass sheets (500 mm × 500 mm × 4.2 mm) were from Guangzhou Jingke Chemical Equipment Co. All chemicals were used as received unless specified otherwise. Deionized (DI) water was used in all experiments.

### 2.2. Functionalization of antimony-doped tin oxide nanoparticles with 3-methacryloxypropyltrimethoxysilane

ATO NPs were added to ethanol/water solution (9.5/0.5, v/v) with a concentration of 10 g/100 ml (with 30 wt% MPS relative to ATO). Then ammonia solution (5 wt%) was added with stirring at 500 r.p.m. to the dispersion to make pH 9.5. MPS surface functionalization of the ATO NPs was performed by adding excessive silane coupling agent, MPS/ATO (0.3 g/1.0 g), directly into the dispersion, followed with ultrasonication for 10 min with continued stirring for 12 h at 70°C with reflux condenser, to promote covalent bonding of the MPS to the surface of the ATO NPs. The hydrolysis of silane methoxy groups, generating Si-OH groups, occurs near the powder surface with the assistance of ultrasonication, just before condensation with single Sn(Sb)-OH groups. The obtained MANs were purified by repeated centrifugation (12 000 r.p.m. for 20 min) and washing with ethanol to remove any excess ammonia and MPS. Finally, the resulting MANs powder was air-dried at 70°C for 24 h prior to being used in the miniemulsion polymerization process.

### 2.3. Miniemulsion polymerization of MPS-functionalized ATO NPs/PMMA-co-BA hybrid latex

Before miniemulsion polymerization, the aqueous phase and the oil phase were separately prepared. The oil phase of MMA, BA and MANs (10 wt%) and hexadecane as co-surfactant was stirred vigorously to allow effective dispersion of the MANs into the monomers. The aqueous phase contained DI water, anionic surfactant SLS and a reactive anionic surfactant (DNS-86, allyloxy polyoxyethylene(10) nonyl ammonium sulfate), which helps to disperse MANs in the nanocomposite [35]. First, the aqueous phase solution was introduced into a 250 ml flask to which the oil phase solution was added slowly over 5 min with vigorous stirring; the stirring was continued for another hour. Subsequently, the miniemulsion was obtained by ultrasonicating the mixture solution for about 30 min with an ultrasonic processor (300 W model SN-30001 Ultrasonicator, Guangzhou Sinochemistry Ultrasonic Equipment Co.) operated at 30% amplitude. During sonication, the emulsion was cooled by ice to avoid any polymerization caused by heating. The resulting miniemulsion was transferred into a thermostated reactor with a condenser and a nitrogen inlet.

**Scheme 1.**
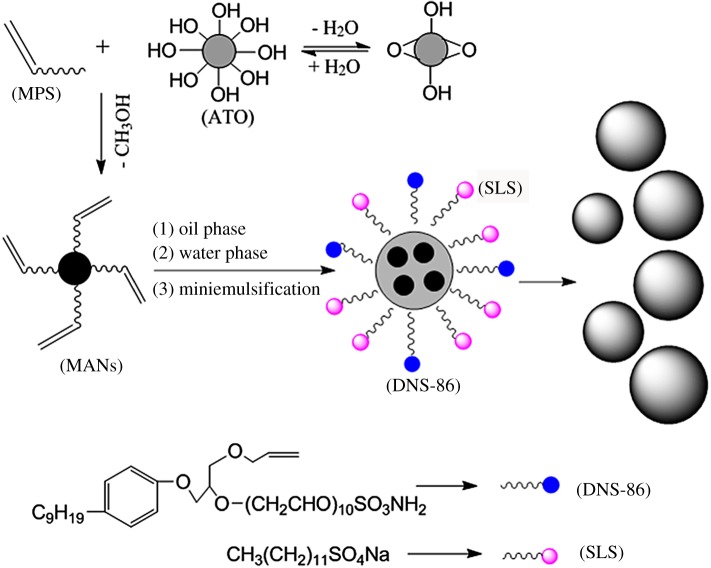
Procedure for the preparation of MANs/PMB nanocomposite latex NPs.

Table 1 shows the constituents used in the preparation of MANs/PMMA-co-BA (MANs/PMB) nanocomposite latex. The polymerization was performed under N_2_ at 70°C and initiated by adding AIBN ethanol solution. The polymerization was finished after 4 h and the reaction was terminated by cooling to ambient condition. As a result, grey-blue latex was obtained. The conversions in all experiments were around 85–90% by mass relative to the initially added non-volatile components (including monomers, MANs and surfactants). MANs/PMB with various MANs contents were prepared by miniemulsion copolymerization of MMA and BA. The process of preparing core-shell structured MANs/PMB nanocomposite latex particles is illustrated in scheme 1.

**Table 1.** Constituents used in the preparation of MANs/PMB nanocomposite latex. Solids content: 21.32%.

components	amount (g)
*oil phase*	
MMA	8.8–11.0^a^
BA	7.2–9.0^a^
MANs or ATO	0–4.0
SLS	0.288
hexdecane	(25 mM)^b^
*aqueous phase*	
DNS-86	0–0.3^c^
deionized water	80
sodium bicarbonate	0.0216

^a^MMA/BA in copolymer: 45/55.

^b^Based on oil phase.

^c^Based on ATO loading: 10%.

### 2.4 Characterization

#### 2.4.1. Transmission electron microscopy

Particle morphologies were observed using JEOL JEM100CXII TEM operated at 80 kV. To prepare samples for transmission electron microscopy (TEM), 0.1% ATO dispersion was obtained by dispersing ATO powder in DI water and sonicating for 30 min; then a drop of ATO dispersion was diluted with 2 ml DI water; a drop of MANs solution or final emulsion was diluted with 2 ml of DI water. A drop of the diluted sample was placed onto a 400 mesh copper grid and dried at room temperature. The number-average particle size of the MANs/polymer plain hybrid polymer particles were determined by counting at least 200 particles. The samples were observed directly without further staining for improved contrast.

#### 2.4.2. X-ray diffraction

X-ray diffraction (XRD) characterizations were carried out on a Rigaku D/Max-IIIC X-ray diffractometer operating at 40 kV with Cu–K*α* radiation (*λ* = 1.5406 Å) in the 2*θ* ranging from 20° to 80°.

#### 2.4.3. X-ray photoelectron spectroscopy

X-ray photoelectron spectroscopy (XPS) data of MANs were collected in both survey and high-resolution modes using a Krafos Axis Ultra DCD system (Rigaku, Japan). The sample was mounted on a standard sample holder and analysed using a micro-focused, monochromated Al K*α* source and operating at 150 W. The scanning scope was 700 × 300 µm. The XPS survey was conducted at a photoelectron emission takeoff angle of 90°. The binding energy of C 1s (284.8 eV) was employed as reference for calibration of binding energy.

#### 2.4.4. Fourier transform infrared spectroscopy

Solutions of ATO, MANs and MANs/PMB in DI water were placed on a potassium bromide (KBr) wafer and dried with an infrared lamp. Fourier transform infrared spectroscopy (FTIR) spectra were obtained in transmission mode using a 300E JASCO FTIR spectrometer (JASCO, Japan). The spectra were collected from 4000 to 500 cm^−1^, with a 2 cm^−1^ resolution over 20 scans.

#### 2.4.5. Particle size and distribution of the nanocomposite latex nanoparticles

Size and size distribution of the latex particles were determined using a dynamic light scattering instrument (ZS Nano S, Malvern Co., UK) at 25°C and at a scattering angle of 90°.

#### 2.4.6. Ultraviolet–visible–near infrared spectroscopy

An ultraviolet–visible–near infrared spectroscopy (UV–Vis–NIR) spectrophotometer (U-4000, Hitachi, Japan) was used to measure the transmittance spectra of the samples from 200 to 2500 nm. MANs/PMB composite films, 10 µm thick, were prepared by casting the hybrid latex on glass sheets or glass slides. The cast films, after 1 day at room temperature, were dried in an oven at 60°C for 20 h. The spectral reflectance and transmittance of the films were determined by integrating the measured spectral data against the Air Mass 1.5 global spectrum using 105 weighted ordinates [36]. Since the sum of the reflectance (*R*), transmittance (*T*) and absorptance (*A*) is one (i.e. *R* + *T* + *A* = 1), the spectral absorptance across the solar spectrum of the samples may be calculated from knowing the *T* and *R* values.

#### 2.4.7. Heat-blocking performance assessment

**Figure 1.**
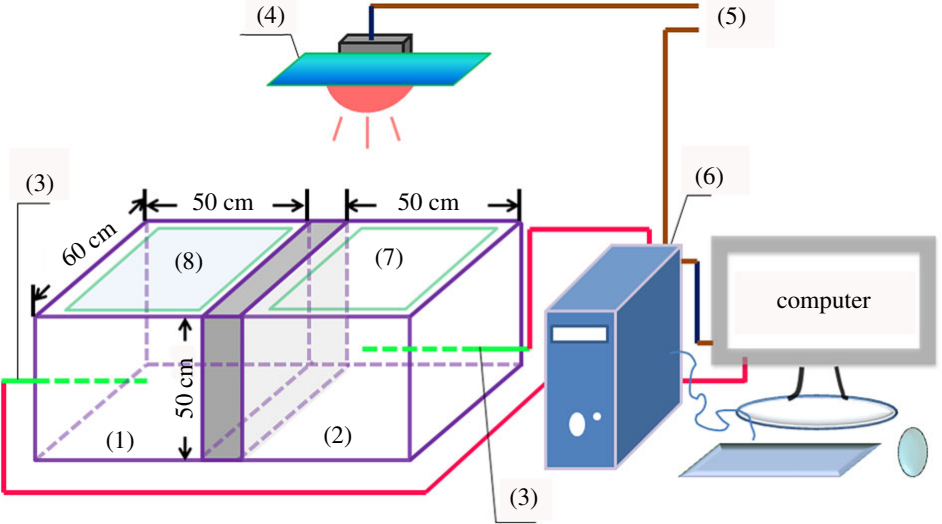
The apparatus for heat-blocking measurement: thermal insulating chamber (1) and (2); thermometer (3); iodine-tungsten light (4); power (5); data collecting system (6); glass coated with PMMA-co-BA (7) and glass coated with MANs/PMMA-co-BA (8).

The heat insulation performance of the composite coatings was determined using an apparatus fabricated for this purpose (figure 1). The apparatus consisted of one iodine-tungsten light fixed on the ceiling, just middle of both chambers, a wooden box with two chambers, each (with a thermocouple fixed in the centre and connected to a data acquisition computer). The illuminated surface of one chamber was painted with PMB and the other with MANs/PMB. The air temperature of the chambers was recorded over time in ambient temperature, about 17°C.

The appearances of the samples were recorded by a digital camera (A640, Canon Inc.). A given amount of MANs was calcinated to 600°C for 4 h, and the grafting percentage of MPS to MANs (*G*%) was calculated as:

2.1 $$G\%=\frac{w_{\mathrm{MANs}}-w_{\mathrm{ATO}}}{w_{\mathrm{MANs}}}\times 100\%,$$

where *w*_MANs_ is the weight of MANs and *w*_ATO_ the weight of ATO.

## 3. Results and discussions

### 3.1. Morphology of nanocomposite latex particles

**Figure 2.**
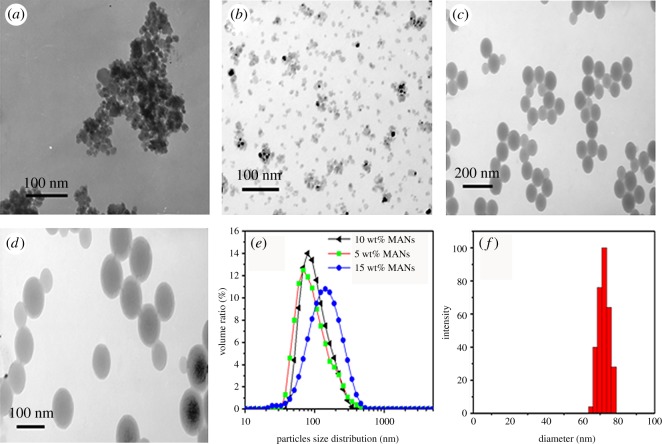
TEM micrographs of ATO NPs (*a*), MANs (*b*), composite latex particles (*c*) and (*d*) with 10 wt% MANs, distribution of latex particles size (*e*), latex particles size with 10 wt% MANs (*f*).

Samples were taken after polymerization for morphology characterization (figure 2). The MAN/PMB nanocomposite particles were in a cluster (figure 2*a*), with individual particles exhibiting a core-shell structure, with MANs core (dark colour) and polymer shell (light colour) (figure 2*c*,*d*). The links between clustering particles are attributed to chemical bonds arising from the functionalization process and polymerizable surfactant, as shown in scheme 1. It is easy for the micelles composed of DNS-86 and SDS to be adsorbed on the miniemulsion droplet surface of the oil phase, and hydrophobic bond was formed between the MPS functionalizations on the MANs surface, monomers and the adsorbed DNS-86. The DNS-86 has been identified as a key to the formation of cluster morphology of the hybrid particles [37]. Because the copolymer has been grafted to the surface of MANs in the miniemulsion droplets, the polymerizable surfactant DNS-86 will be initiated by the macromolecule radical group at the droplet interfaces and form a bigger radical group during the polymerization. Subsequently, the radical group of the droplets interact with each other, which eventually leads to the formation of hybrid latex with core-shell structure.

TEM micrographs show that the MANs were encapsulated during the polymerization of MMA and BA with the evident formation of PMMA-co-BA (PMB) coating. Also the average size of MANs/PMB particles was around 70 nm (figure 2*e*), which is in good agreement with that observed with TEM. It should be noted that no free MANs were found in the hybrid latex, suggesting good dispersion of MANs (figure 2*b*). Then monomer droplets containing MANs have been dispersed in the aqueous phase and stabilized by surfactant SDS and co-surfactant and embedded in latex by reacting with monomers during polymerization. The migration of MANs from stabilized mini droplets hardly occurs and those that come out could not maintain their dispersibility and will aggregate and precipitate during the progression of miniemulsion polymerization. It means that almost all MANs are embedded in a stable core-shell latex composite.

### 3.2. Stability of the nanocomposite latex

Owing to the importance of surface functionalization of the NPs with organic group, MANs were synthesized with excess MPS by adapting reported methods (figure 2*b*) [38]. The morphology of the MANs clearly shows layering (figure 2*b*). This is attributed to functionalization of the hydrophilic ATO surface with MPS, which renders the particles hydrophobic, while retaining their colloidal stability in organic solvents as previously reported [39].

**Figure 3.**
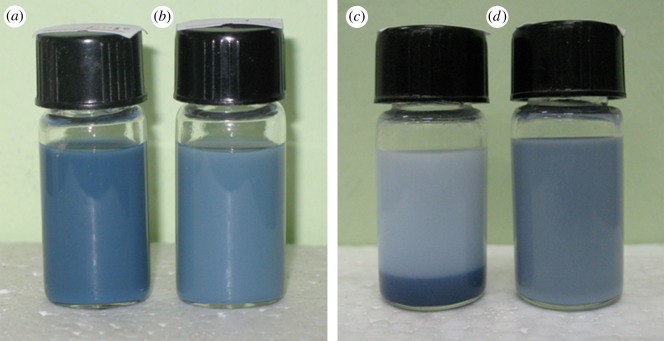
MANs (*a*) and ATO (*b*) in ethanol/water solution, ATO/PMB nanocomposite latex (*c*) and MANs/PMB nanocomposite latex (*d*) in water solution.

The experimental results show when unfunctionalized ATO NPs are used, the polymer cannot form strong interaction with them and they will settle down in solutions of pH approximately 6.5 [40] (figure 3). From the appearance of ATO dispersions in ethanol/water solution functionalized with MPS (figure 3*a*) and without (figure 3*b*), it can be seen the colour of the dispersion becomes darker after being functionalized with MPS (figure 3*a*). The reason might be that the ATO aggregates are broken into nanosized particles, and the nanosize effect causes the reflecting light to move to shorter wavelength, as shown in figure 3*a* [41]. The MPS grafting rate *G*% is about 9.6%, as calculated by equation (2.1). It is also said that about two-thirds of the total MPS did not graft to the surface of ATO nanoparticles. Such a low *G*% may relate to the low amount of surface hydroxide.

This might be the reason why the latex composite prepared with unmodified ATO NPs was unstable (figure 3*c*). ATO NPs precipitated and settled on the bottom of the bottle after allowing to stand for 5 h (figure 3*c*). However, due to the presence of lots of anchor sites on the surface of MANs, the polymer can be grafted to form a strong covalent bond. MANs can be encapsulated in the polymer latex particles, forming core-shell co-structure and the appearance of the latex becomes grey-blue (figure 3*d*). So the latex is stable without any settling of MANs that can be seen obviously after 5 days (figure 3*d*), and remained so even after allowing to stand for 30 days.

### 3.3. Fourier transform infrared spectroscopy analysis

**Figure 4.**
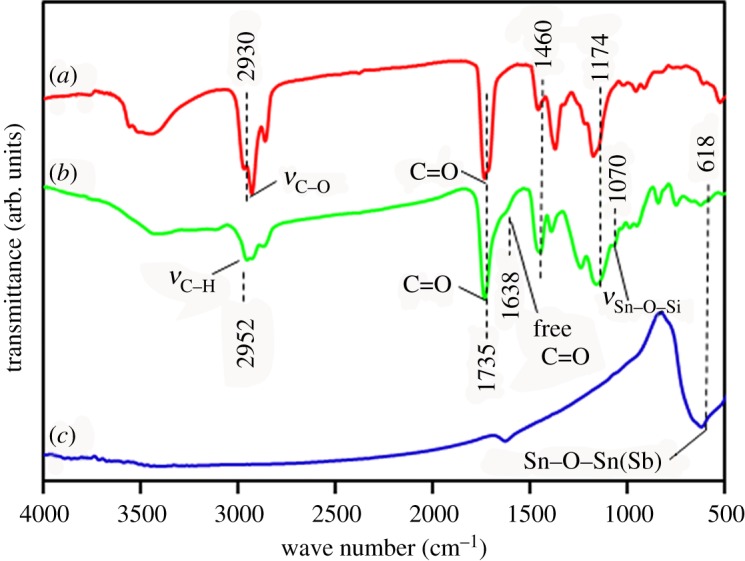
FTIR spectra of ATO NPs (*a*), MANs/PMB nanocomposite latex(*b*) and PMB latex (*c*).

The FTIR spectra of ATO, MANs/PMB and PMB are shown in figure 4*a*–*c*, respectively. The characteristic bands belonging to PMB chains at 2930, 1735, 1460 and 1174 cm^−1^ in the adsorption peaks are shown in figure 4*b*,*c*, among which the in-plane vibrations of methylene group C–H shifts from 2930 cm^−1^ [42] to 2952 cm^−1^. Such a shift might point to the induction effect between the oxygen atoms of ATO and PMMA-co-BA. However, the peaks that appeared at 1460 and 1174 cm^−1^, respectively, referred to the bending vibration peak of C–H in acrylic copolymer and the asymmetric stretching vibration of Si–O–Sn, which are the specific bands of the ATO nanoparticles [43,44].

The broad complex band in the region of 710–500 cm^−1^ corresponds to the asymmetric stretching vibrational mode of the Sn–O–Sn bridge in the MANs [45]. Figure 4*b* has an extra peak at 1070 cm^−1^, which is due to Si–O–Sn vibrations of MPS. A small shoulder visible at 1638 cm^−1^ also attests to the presence of C=O and is attributed to the free carbonyl groups that are not involved in hydrogen bonding, as previously reported [40]. These peaks, present in figure 4*b*, indicate further the reaction between MPS and ATO NPs. All the results indicate the successful incorporation of MANs in the copolymer chains via miniemulsion polymerization with MMA and BA.

### 3.4. X-ray photoelectron spectroscopy analysis of MPS-functionalized ATO NPs/PMMA-co-BA

**Figure 5.**
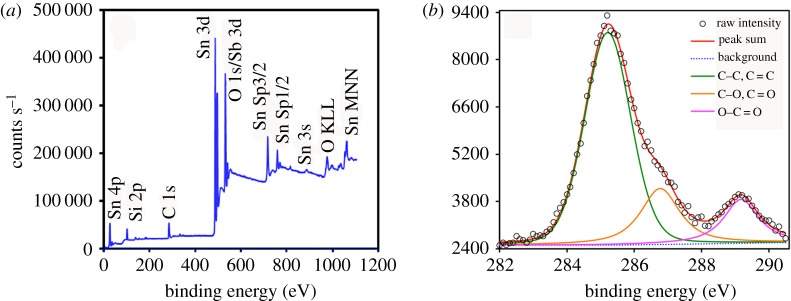
Survey (*a*) and C 1s XPS spectra (*b*) of MANs.

To further confirm the existence of the MPS-functionalized ATO NPs in the hybrid particles of MANs/PMB, XPS mapping was used to investigate the surface structure of MANs with an axis ultramulti-technique electron spectrometer with an Al K*a* X-ray source and operating at 150 W, as shown in figure 5. The O 1s window shows two peaks with similar areas at 530.8 ± 0.1 and 531.2 ± 0.1 eV, respectively, corresponding to the two different types of oxygen in the ester functional group or Sb 3d, as shown in figure 5. There is also a peak of Si 2p which corresponds to Si–O at 102.1 eV [46,47]. The C 1s window shows a complex pattern of peaks which stand for three kinds of carbon bonds (289.2 eV may be attributed to C=O, C–O at 286.7 eV and C–C at 285.2 eV, shown in figure 5*b*). As a result, the MPS group has been successfully grafted to the surface of ATO NPs. This also indicates the successful incorporation of MANs in the copolymer latex.

Also, it indicated MPS bonded successfully to the surface of ATO, in good agreement with the literature. As reported [40], the hydroxyl groups on the NPs surface react with MPS at temperatures between 400 and 1100°C. According to the report, achieving such high temperatures required for the reaction is usually very difficult. However, it will become possible with the aid of ultrasonication. It was reported that ultrasonication of a liquid caused the formation, growth and implosive collapse of bubbles, which could generate hot spots of temperatures approximately 5200 K [45]. The low-temperature NPs surface functionalization reaction that happens here is from the ultrasonic stirring facilitating a local high temperature near the NPs surface.

### 3.5. X-ray diffraction analysis

**Figure 6.**
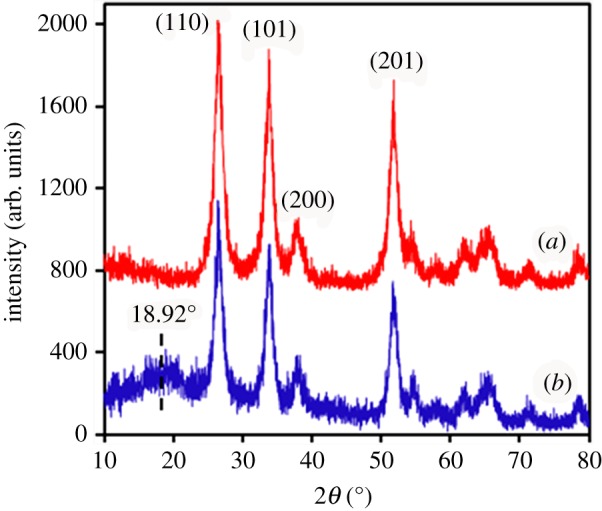
XRD patterns of ATO (*a*) and MANs/PMB nanocomposite latex (*b*).

Figure 6 shows the XRD patterns of ATO and MANs/PMB latex NPs, which indicate that the peak positions of both ATO and MANs/PMB are in good agreement with the standard data (JCPDS Card No. 77-0452). The particles possessed a cassiterite type with a tetragonal rutile structure. The XRD patterns of both show sharp peaks at (110), (101) and (201). Compared with figure 6*a*, the intensity of these peaks decreased with the presence of the copolymer in the composites in figure 6*b*. Moreover, it was observed that the basic peaks of ATO were intact in the composite of MANs/PMB, indicating that no obvious changes were imparted during the preparation of the MANs/PMB composite, nor were the relative peak positions shifted significantly due to the presence of the polymer. However, the MANs/PMB nanocomposite shows a broad peak at 2*θ* = 18.92°, due to the copolymer bonded with the MANs, shown in figure 6*b*. In addition, the PMMA-co-BA in the nanocomposite sample was amorphous; therefore, no obvious crystallite was observed, as shown in figure 6*b*. This again indicated the MANs were successfully incorporated in the copolymer latex.

### 3.6. The transparency and heat-blocking effects

The transparent heat insulation coatings with 5 and 10% weight contents of MANs were sprayed on dried glass slides. The thickness of coated film was about 10 µm. One slide coated with pure poly(MMA-co-BA) (PMB) latex, synthesized by the same process of miniemulsion polymerization, was used as control.

**Figure 7.**
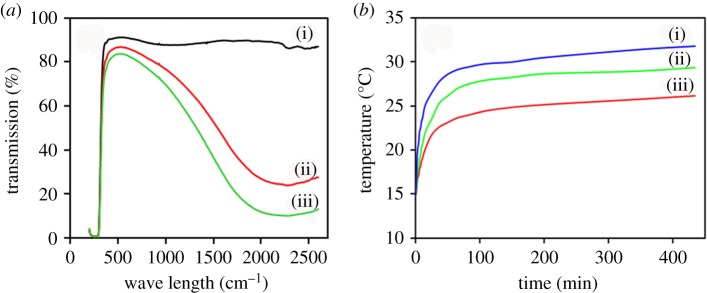
(*a*) Transmission of glass slides cast with MANs/PMB nanocomposite latex: (i) 0, (ii) 5 and (iii) 10, (*b*) MANs/PMB nanocomposite latex coating glass sheets: (i) 0, (ii) 5 and (iii) 10.

The UV–Vis–NIR transmission (spectral range = 0.2–2.5 µm) of the glass slides are shown in figure 7*a*. The transmittances of glass coated with MANs/PMB containing 0%, 5% and 10% of MANs were about 91.0%, 86.5% and 83.2%, respectively. However, the transmission in the NIR region (780–2500 nm) decreased significantly, roughly more than 50% compared to in the visible region. That is, glass coated with MANs/PMB containing 10% MANs can shield more NIR light, about 70%.

The heat insulation properties of the ATO-functionalized with MPS (MANs)/PMB composite latex are presented in figure 7*b*. Under the iodine-tungsten light, heat insulation performance assessment was carried out with a two-chamber device, as shown in figure 1. The chamber temperature gradually increased with time and then reach a steady state within around 6 h, as shown in figure 7*b*. In the chamber with MANs/PMB-coated glass sheets, temperature increased more slowly than that with PMB-coated glass sheet. The highest difference between the temperatures in chambers with MANs/PMB-coated (5 wt% MANs) glass and with PMB-coated glass is about 3.3°C. With increasing MANs content in MANs/PMB composite, the highest temperature difference also increased. For the MANs/PMB with 10 wt% MANs, the temperature difference is up to about 6.5°C (figure 7*b*). Meanwhile, in good agreement with the results of other studies [34,48], the transmittance of the MANs/PMB nanocomposite decreases as the weight content of MANs increases. Owing to the precisely tailored core-shell structure, the nanocomposite film shows a more excellent heat-blocking property than that reported in the literature [34,48]. These results indicate that the MANs/PMB nanocomposite latex has good heat-blocking properties and can be used in windows of buildings and vehicles and other thermal management applications.

## 4. Conclusion

We synthesized MANs/PMB nanocomposite latex NPs with core-shell structure via miniemulsion polymerization of MMA and BA with MPS-functionalized ATO NPs (MANs). The nanocomposite films we synthesized can transmit over 80% in the visible region and cut off more than 50% of NIR rays at the same time. Therefore, the MANs/PMB nanocomposite latex we prepared has good heat-blocking properties. It makes the nanocomposite latex of MANs/PMB a promising candidate for sun-shading applications as well as transparent thermal insulating coating material, for use in energy-saving windows, windshields of cars, etc.

## Data accessibility

All of the data in this investigation have been reported in the paper and are freely available.
